# Efficacy of remimazolam with fentanyl vs midazolam with fentanyl for sedation in screening colonoscopy: Randomized controlled study

**DOI:** 10.1055/a-2655-1083

**Published:** 2025-08-15

**Authors:** Andrea C Armbrecht, Bojan Kovacevic, Maria Dyrehave Rasmussen, Michelle Katharina Bernth, Ann Merete Moeller, Peter Vilmann

**Affiliations:** 1 53176 Herlev, Herlev Hospital, Herlev, Denmark; 2 27983 Antioquia, Universidad de Antioquia, Medellin, Colombia; 3 Gastro Unit, Division of Endoscopy, Copenhagen University Hospital Herlev, Herlev, Denmark; 4 53212 Medicinsk afdeling, Dronning Ingridip Napparsimmavissua, Nuuk, Greenland; 5 53139 SUND, Københavns Universitet Det Sundhedsvidenskabelige Fakultet, Kobenhavn, Denmark; 6 Anaesthesia, Copenhagen University Hospital Herlev, Herlev, Denmark

**Keywords:** Endoscopy Lower GI Tract, CRC screening, Colorectal cancer, Endoscopic resection (polypectomy, ESD, EMRc, ...)

## Abstract

**Background and study aims:**

Remimazolam is a new ultra-short-acting benzodiazepine with a favorable safety-profile when used for sedation in endoscopy. The aim of this project was to investigate efficacy of remimazolam with fentanyl compared with midazolam with fentanyl for sedation in colonoscopy among fecal immunochemical test (FIT)-positive screening participants.

**Patients and methods:**

The study was a prospective, single-blinded, randomized controlled trial. FIT-positive participants undergoing colonoscopy were randomized to intravenous remimazolam + fentanyl (RF) or midazolam + fentanyl (MF). Primary outcome was total time from start of medication until discharge. Secondary outcomes included time to reach cecum, need for post-procedure recovery, patient-reported pain and satisfaction, need for additional medication, and procedure completion.

**Results:**

A total of 205 patients were included and randomized 1:1 (RF:103, MF:102). Mean age was 62.6 years, whereas female/male ratio was 97/108. Mean time from start of medication until discharge was 29.9 minutes (RF) versus 35.0 minutes (MF) (95% confidence interval 0.77–0.94, *P* = 0.012). Mean time to reach the cecum was 15.4 minutes (RF) compared with 20.2 minutes (MF) ( *P* = 0.001). Proportion of patients requiring postoperative observation was lower for RF 0.97% vs 9.8% for MF ( *P* = 0.022). Patients receiving remimazolam reported an average lower pain score (mean 2.25 (RF) vs 3.25 (MF) *P* = 0.012) and higher overall satisfaction score (4.65 vs 4.33, *P* = 0.012).

**Conclusions:**

This study shows clear superiority of the combination of remimazolam with fentanyl over midazolam with fentanyl for conscious sedation in screening colonoscopy, obtaining shorter procedure time, less postoperative need for observation, lower patient pain scores, and higher patient satisfaction.

## Introduction

Colorectal cancer is the third most common cancer globally, with the second-highest mortality worldwide, with more than 1.9 million new cases and 935,173 deaths reported worldwide in 2021 1 . In Denmark, all men and women aged 50 to 74 are invited biannually to participate in screening for colorectal cancer 2 , which consists of a fecal immunochemical test (FIT) followed by a colonoscopy if the result is positive. Screening is recommended to detect cancer at an early stage, allowing for milder treatment, a higher chance of cure, and a reduction in cancer incidence and associated mortality 2 3 4 . Unfortunately, colonoscopy can be unpleasant and painful, and in most cases, requires sedation to increase both the comfort and quality of the procedure 5 6 . A combination of intravenous (IV) midazolam and fentanyl or propofol sedation is commonly used, although both have disadvantages. Propofol has a quick onset and offset, which allows for a rapid turnover of cases but is more strongly associated with hypoxia, hypotension, and bradycardia 7 . Midazolam sedation results in more stable hemodynamic responses 8 but may result in delayed discharge due to prolonged elimination 9 .

Remimazolam is a new ultra-short acting benzodiazepine, and although, like midazolam, it is a GABA receptor agonist, it does not rely on cytochrome p450 for its metabolism but is instead metabolized through tissue esterases. Therefore, its distribution half-life is much shorter—0.5 to 2 minutes compared with 4 to 18 minutes for midazolam—and its terminal elimination half-life is only 7 to 11 minutes (vs 1.7 to 2.4 hours for midazolam) 10 . Recent studies have demonstrated the noninferiority of remimazolam sedation to propofol sedation in terms of discharge time after colonoscopy 11 12 . Furthermore, remimazolam was associated with less injection pain and a lower risk of hypotension and bradycardia 13 . Superiority of remimazolam over midazolam and placebo was also demonstrated in a randomized controlled study (RCT), showing faster recovery and discharge of patients undergoing colonoscopy 14 .

The overall objective of this study was to investigate whether a combination of remimazolam and fentanyl is superior to midazolam with fentanyl for sedation in screening colonoscopy, and to examine whether remimazolam improves procedure sedation, procedure time, and recovery time.

## Patients and methods

### Study design and patients

This was a prospective, single-blinded, RCT with two parallel groups and a 1:1 allocation ratio adhering to the standards of good clinical practice (GCP). FIT-positive screening participants scheduled for colonoscopy at the Endoscopy Unit, Herlev and Gentofte Hospital, were assessed for eligibility and included upon providing written informed consent. Patients who declined sedation, required deep sedation, or had contraindications to remimazolam or midazolam were excluded. All data collection and handling were performed in accordance with the General Data Protection Regulation (GDPR) and the study was approved by the regional ethics committee (No. p-2023–14227) and registered at Clinical Trials in the European Union - EMA (EudraCT No: 2023–503470–21–00).

### Interventions

Sedation was administered by nurses trained in conscious sedation under the direct supervision of endoscopists. Blood pressure, heart rate, oxygen saturation, and respiratory frequency were monitored continuously during the procedure until the discharge criteria were fulfilled. Continuous oxygen therapy was administered through a nasal catheter during sedation.

All subjects, regardless of their assigned group, initially received a standard dose of 50 µg fentanyl. Patients randomized to the control group (MF) received an induction dose of 2 mg of midazolam and 1 mg as an additional dose, if needed, up to a maximum of 4 mg. Patients randomized to the experimental group (RF) received an IV induction dose of 5 mg remimazolam and 1.25 to 2.5 mg as an additional dose when necessary, up to a maximum of 20 mg. Reduced induction and maintenance doses were used in elderly patients and/or patients with a low body mass index. Colonoscopy was initiated when adequate sedation was reached and assessed using the Modified Observer's Assessment of Alertness and Sedation (MOAA/S) score 15 . This scale ranges from 0 to 5, with a score of 5 defined as awake or minimally sedated and a score of 0 defined as general anesthesia ( **Supplementary Table 1** ). A MOAA/S score ≤ 4 was accepted as adequate sedation. The score was assessed every 5 minutes following initial sedation, and if necessary, additional sedation was administered until the cecum was reached. Pain, despite the initial dose of fentanyl, was treated with supplementary doses of 25 to 50 µg fentanyl, up to a maximum of 150 µg.

Colonoscopies were performed by gastroenterology or gastrointestinal surgery consultants and polypectomies were performed during withdrawal.

Adverse events (AEs) during sedation were defined according to the AGREE classification of AEs in gastrointestinal endoscopy 16 . In addition, changes in vital signs not otherwise classified as AEs were defined as incidences: hypoxemia (oxygen saturation < 92%), hypotension (systolic blood pressure (BP) < 80 mm Hg and/or diastolic BP < 40 mm Hg), hypertension (systolic BP > 180 mm Hg and/or diastolic BP > 100 mm Hg), bradycardia (heart rate < 50 bpm), and tachycardia (heart rate > 100 bpm).

Following completion of the procedure, the patients were assessed using the official discharge criteria at our department ( **Supplementary Table 2** ). A score of 1 or 0 indicated that the patient could be discharged. Otherwise, the patient was transferred to the recovery room, where regular monitoring was performed according to the aforementioned criteria.

### Outcomes

The primary outcome was the total time from start of medication to fulfillment of discharge criteria ( **Supplementary Table 2** ), which included level of consciousness/alertness, respiratory frequency, and blood oxygen saturation compared with preprocedure baseline.

Secondary outcomes were: procedure completion (reaching the cecum and ability of confirming/excluding colon cancer on retraction), time to reach the cecum, endoscopist satisfaction (on a 5-point Likert scale), need for additional fentanyl and sedation, need for a recovery room, and patient discomfort or pain using the Numeric Rating Scale 11 (NRS-11). Furthermore, patient satisfaction was measured through questionnaires on a 5-point Likert scale measuring satisfaction with amount of sedation, experienced side effects, and general satisfaction. Questionnaires were handed out immediately after the procedure and repeated after 2 days via email or over the phone.

### Randomization and blinding

Patients who met all inclusion and exclusion criteria were randomly assigned to either the control or experimental group with a 1:1 allocation ratio on the day of the procedure using a computer-generated allocation table. The concealed allocation table was incorporated into a secure online data repository (REDCap) and randomization was performed directly in this system by a research team member not involved in the inclusion or assessment of outcomes. Patients were blinded to the allocation, but due to different dosage regimens, it was not possible to blind the endoscopy staff or investigators present during the colonoscopy.

### Statistical analysis and sample size

Sample size was determined through multiple simulations (n = 1000) based on assumptions of a median discharge time of 35 minutes in the control group and a 10% decrease in the intervention group. The dispersion parameter was estimated based on the historical procedure data. At 80% power and a significance level of 0.05, a minimum of 200 patients were required.

Continuous data were summarized as means with standard deviations or medians with minimum and maximum values. Generalized linear models were used to compare time until the cecum was reached and total time until discharge (negative binomial regression). Satisfaction and pain scores were compared using the Wilcoxon rank-sum test, whereas Fisher’s exact test was used to compare procedure completeness, need for a recovery room, and need for supplementary medication. AEs and procedure incidences were compared using the Fisher’s exact test.

Given multiple comparisons among secondary outcomes, the Benjamini-Hochberg procedure was applied to adjust *P* values, controlling the false discovery rate at 0.05. Adjusted *P* values below this threshold were considered statistically significant. All statistical calculations were performed using the R statistical program (version 4.4.2). All authors had access to the study data and reviewed and approved the final manuscript.

## Results

**Fig. 1:**
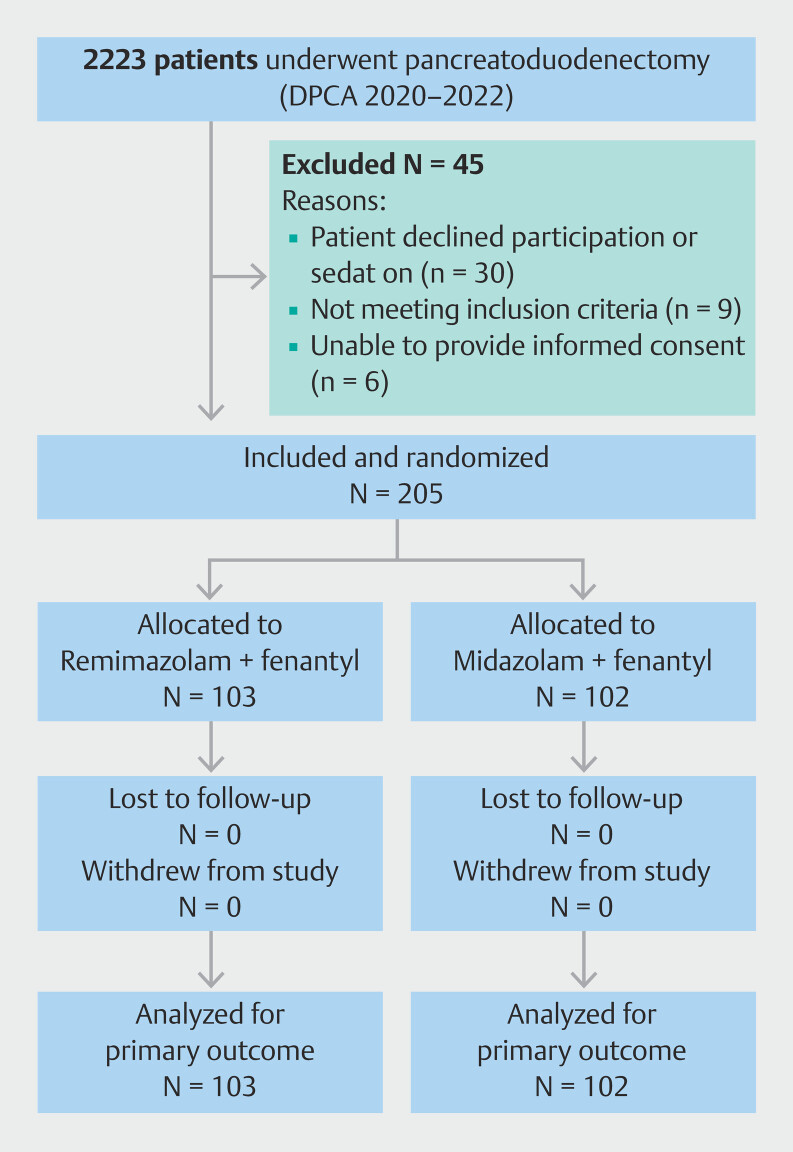
CONSORT flow diagram 17 .

We assessed 250 patients for eligibility between August and December 2023, of whom 45 were excluded because they did not meet the inclusion criteria ( Fig. 1 ). Of the 205 patients included, 103 were randomized to the RF study arm (intervention group) and 102 to the MF study arm (control group). Mean age of patients was 62.75 years (SD 7.7), with a female/male ratio of 97/108. Baseline data for the patients are presented in Table 1 .

**Table:** **Table 1** Baseline data for the two groups.

	Control group (midazolam + fentanyl) (N = 102)	Intervention group (remimazolam + fentanyl) (N = 103)	Overall (N = 205)
**Age (years)**			
Mean (SD)	62.7 (7.64)	62.8 (7.78)	62.8 (7.69)
**Gender**			
Women	51 (50.0%)	46 (44.7%)	97 (47.3%)
**Body mass index**			
Mean (SD)	26.7 (4.48)	27.3 (5.72)	27.0 (5.14)
**ASA score**			
I	77 (75.5%)	80 (77.7%)	157 (76.6%)
II	21 (20.6%)	20 (19.4%)	41 (20.0%)
III	2 (2.0%)	3 (2.9%)	5 (2.4%)
NA	2 (2.0%)	0 (0%)	2 (1.0%)
**History of diabetes**			
Yes	6 (5.9%)	8 (7.8%)	14 (6.8%)
**History of cardiovascular disease**			
Yes	22 (21.6%)	28 (27.2%)	50 (24.4%)
**Previous surgery**			
Pelvic/abdominal surgery	38 (37.3%)	38 (36.9%)	76 (37.1%)
No previous surgery	43 (42.2%)	44 (42.7%)	87 (42.4%)
**Endoscopic experience (years)**			
Mean (SD)	11.7 (8.8)	11.7 (8.5)	11.7 (8.6)
ASA, American Society of Anesthesiologists; SD, standard deviation.			

### Primary outcome

**Fig. 2:**
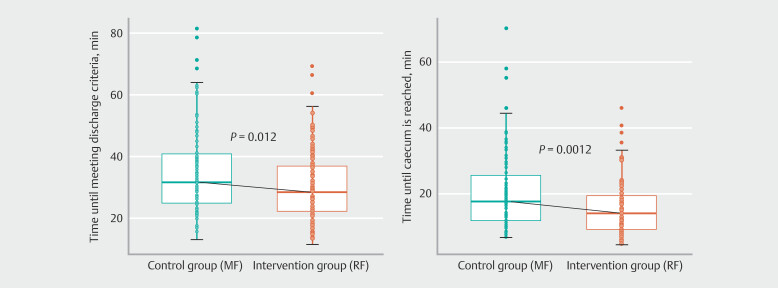
Time until discharge criteria were met (left) and time until cecum was reached (right) in the two groups.

Mean total time from start of medication to meeting discharge criteria was 29.9 minutes in the remimazolam group, compared with 35.0 minutes in the control group. The estimated ratio between the groups was 0.85, corresponding to a 15% reduction in total time in the remimazolam group, reaching statistical significance (95% CI 0.77–0.94, *P* = 0.012) ( Fig. 2 ).

### Secondary outcomes

Secondary outcomes are presented in Table 2 and Table 3 . The rate of procedure completion was 99% in both groups. Time to reach cecum was shorter in the RF group (mean 15.4 min, median 13.9 min) compared with the MF group (mean 20.2 min, median 17.6 min) ( *P* = 0.0012). Need for a recovery room was lower in the RF group (0.97%), compared with the MF group (9.8%) ( *P* = 0.022). Patients in the RF group experienced less pain on the NRS-11 scale (RF: mean 2.25, median 1.0 vs MF: mean 3.25, median 3.0; *P* = 0.012).

**Table:** **Table 2** Primary and secondary outcomes and other variables.

**Variable**	** Control group (midazolam + fentanyl) ** (N = 102)	** Intervention group (remimazolam + fentanyl) ** (N = 103)	***P* value **
**Procedure completion**			
Complete	101 (99.0%)	102 (99.0%)	1.000
**Total time from start of medication until cecum reached (min)**			
Mean (SD)	20.2 (11.4)	15.4 (8.37)	
Median [min, max]	17.6 [6.63, 70.0]	13.9 [4.48, 46.0]	0.0012
NA	1 (1.0%)	1 (1.0%)	
**Endoscopist satisfaction with sedation**			
1 (very dissatisfied)	0 (0%)	0 (0%)	
2 (dissatisfied)	3 (2.9%)	3 (2.9%)	
3 (neutral)	11 (10.8%)	5 (4.9%)	
4 (satisfied)	22 (21.6%)	21 (20.4%)	
5 (very satisfied)	65 (63.7%)	74 (71.8%)	0.478
NA	1 (1.0%)	0 (0%)	
**Need for supplementary fentanyl**			
Yes	28 (27.5%)	22 (21.4%)	
No	74 (72.5%)	81 (78.6%)	0.399
**Total fentanyl administered**			
< 100 µg	74 (72.5%)	86 (83.5%)	
≥ 100 µg	28 (27.5%)	17 (16.5%)	0.098
**Amount of sedation administered (mg)**			
Mean (SD)	3.14 (0.778)	9.44 (3.25)	
Median [Min, Max]	3.00 [1.00, 5.00]	10.0 [3.75, 20.0]	
**Mean MOAA/S score during first 10 mins**			
Mean (SD)	4.15 (0.426)	3.50 (0.666)	
Median [min, max]	4.00 [2.50, 5.00]	3.50 [1.50, 4.50]	< 0.001
**Need for recovery room**			
Yes	10 (9.8%)	1 (1.0%)	
No	92 (90.2%)	101 (98.1%)	0.0218
NA	0 (0%)	1 (1.0%)	
**Polypectomy**			
Polypectomy performed	45 (45.5 %)	57 (58.1%)	0.125
Total number of polyps removed	88	136	
Cold snare polypectomy	76	127	
Hot snare polypectomy including EMR	12	9	
EMR, endoscopic mucosal resection; MOAA/S, Modified Observer's Assessment of Alertness and Sedation; SD, standard deviation.			

**Table:** **Table 3** Patient satisfaction questionnaires.

	**Procedure day questionnaire**			**Follow-up questionnaire**		
	** Control group (midazolam + fentanyl) ** (N = 102)	** Intervention group (remimazolam + fentanyl) ** (N = 103)	***P* value **	** Control group (midazolam + fentanyl) ** (N = 102)	** Intervention group (remimazolam + fentanyl) ** (N = 103)	***P* value **
**Patient-reported pain on NRS-11 scale**						
Mean (SD)	3.25 (2.73)	2.25 (2.56)		2.52 (2.57)	1.86 (2.53)	
Median [min, max]	3.00 [0, 10.0]	1.00 [0, 9.50]	0.012	2.00 [0, 9.00]	1.00 [0, 10.0]	0.013
NA				6 (5.9%)	7 (6.8%)	
**Patient satisfaction with amount of sedation**						
Mean (SD)	4.23 (1.11)	4.53 (0.872)		4.42 (0.879)	4.67 (0.735)	
Median [min, max]	5.00 [1.00, 5.00]	5.00 [1.00, 5.00]	0.046	5.00 [2.00, 5.00]	5.00 [1.00, 5.00]	0.046
NA				6 (5.9%)	7 (6.8%)	
**Patient satisfaction with amount of nausea and dizziness after procedure**						
Mean (SD)	4.25 (1.07)	4.51 (0.850)		4.25 (0.973)	4.49 (1.01)	
Median [min, max]	5.00 [1.00, 5.00]	5.00 [1.00, 5.00]	0.064	5.00 [1.00, 5.00]	5.00 [1.00, 5.00]	0.018
NA				6 (5.9%)	7 (6.8%)	
**Patient satisfaction with amount of time under effect of medication after procedure**						
Mean (SD)	4.25 (0.930)	4.57 (0.777)		4.32 (0.970)	4.46 (0.972)	
Median [min, max]	5.00 [1.00, 5.00]	5.00 [2.00, 5.00]	0.012	5.00 [1.00, 5.00]	5.00 [1.00, 5.00]	0.235
NA	0 (0%)	1 (1.0%)		7 (6.9%)	7 (6.8%)	
**Patient overall satisfaction with sedation**						
Mean (SD)	4.33 (0.958)	4.65 (0.737)		4.44 (0.960)	4.63 (0.798)	
Median [min, max]	5.00 [1.00, 5.00]	5.00 [2.00, 5.00]	0.012	5.00 [1.00, 5.00]	5.00 [1.00, 5.00]	0.216
NA				6 (5.9%)	7 (6.8%)	
SD, standard deviation.						

We found higher patient satisfaction with the amount of sedation, levels of nausea and dizziness experienced, duration of sedation effects, and higher overall satisfaction in the RF group than in the MF group following the procedure ( Table 3 ), all differences which reached statistical significance. In a follow-up survey, patients in the RF group were generally more satisfied with levels of pain, nausea, and dizziness experienced after the procedure than those in the MF group ( Table 3 ). Patient satisfaction with the duration of sedation effects and overall satisfaction remained higher in the RF group, although the difference did not reach statistical significance.

### Adverse events

No AEs were recorded in either group, according to the AGREE classification of AEs in gastrointestinal endoscopy. Seventeen procedure incidences were observed in total, with nine in the MF group and eight in the RF group ( *P* = 0.853). In all cases, the events were of short duration, with a return to baseline without any additional pharmacological interventions. No patient had more than a single incidence.

## Discussion

In the present study, total time from start of medication until discharge criteria were met was statistically significantly shorter by a mean of 5.1 minutes in the RF group than in the MF group, reaching statistical significance. Similarly, total time from start of medication until the cecum was reached was shorter by a mean of 4.8 minutes in the RF group than in the MF group, which was also statistically significant. We attribute this difference to rapid onset of the sedative effect of remimazolam, which facilitates quick achievement of an appropriate depth of sedation. Depending on the number of endoscopies performed per year, this could translate into a significant time improvement, allowing for a higher turnover as well as an increased number of endoscopies. Interestingly, the proportion of patients in whom a polyp was observed (polyp detection rate), as well as the total number of polypectomies performed, was, on average, higher in the RF group than in the control group. Although this difference was statistically insignificant, it highlights the potential superiority of remimazolam, because polypectomies tend to increase procedure time. Appropriately powered studies should examine the effects of remimazolam on adenoma detection rate and average duration of polypectomy.

Most secondary outcomes favored the RF group ( Table 3 ), with no variables favoring the MF control group. We found a clear difference in patient satisfaction scores in terms of both pain and side effects, as well as overall satisfaction. This finding could be clinically significant, because a less unpleasant experience may result in improved compliance with colonoscopy and subsequent health benefits. The fact that patient discomfort and pain (NRS-11) scores were also lower in the RF group further underscored these benefits. These statistically significant differences were withheld for most variables in the follow-up questionnaires, except for patient satisfaction with the amount of sedation. Similarly, patient subjective perception of time under the effects of sedation was shorter in the RF group than in the MF group. In addition to higher patient satisfaction scores, we observed less need for a recovery room when remimazolam was used. This finding could have practical implications in an endoscopy suite, freeing up resources used in the recovery area and possibly translating into even higher patient turnover. Finally, both sedation regimens were equally safe, with no AEs recorded according to the AGREE classification.

Our results are in line with other clinical trials, such as Dao et al. 10 , who concluded in a post hoc integrated analysis of three clinical trials that there is a clear advantage of remimazolam compared with midazolam in providing faster recovery time and less fentanyl requirement. Although we administered the same dosage of remimazolam as Dao et al and Rex et al 14 (5 mg bolus plus 2.5 mg for maintenance), our study failed to show a reduced need for supplementary fentanyl. We observed an overall tendency for the MF group to require higher fentanyl doses than the RF group, but our study did not have the required power to detect a statistical difference. Nonetheless, the decision to administer an additional dose of fentanyl was made at the discretion of the endoscopist, with individual preferences and variations in what was considered acceptable patient discomfort.

Remimazolam has also been compared with propofol in elderly patients undergoing gastrointestinal endoscopy, and Guo et al 18 found a lower incidence of hemodynamic events and respiratory depression when using remimazolam. The number of supplemental doses was also slightly increased in this group. In our study, we found no difference in AEs between the two groups, demonstrating the excellent safety profile of remimazolam. Similarly, Yueyang Xin 13 studied remimazolam and alfentanil during colonoscopic polypectomy and reported an optimal sedative effect of remimazolam, with a lower incidence of hypotension and, therefore, a safer profile than propofol and alfentanil.

In our study, remimazolam was noted to have certain advantages over midazolam during colonoscopy. First, remimazolam induced a much faster and deeper sedation effect, as well as faster recovery toward MOAA/S 5, so that patients reached ≤ MOAA/S 4 faster, and an average MOAA/S score of 3.5 was achieved during the first 10 minutes of the procedure. This was observed as a clear advantage, enabling the endoscopist to advance through the painful segments more easily. In addition, patients were able to cooperate without significant delay when a position change was necessary. Moreover, after reaching the cecum, patients were awake again and could partake in the endoscopist’s explanation of the findings when deemed appropriate. However, disadvantages of remimazolam seem to be the increased number of doses needed throughout the procedure, requiring frequent attention from the nurse who performed the sedation.

The strengths of our study are that it was a randomized GCP monitored study, carefully planned, and conducted using thorough methodology. There were no patient dropouts or missing data for the primary outcome, with a limited number of missing data in general. In terms of limitations, it should be noted that the study was single-blinded, mainly due to different drug administration regimens. Because the endoscopists were not blinded to the allocation, physician satisfaction with sedation may have been prone to bias. Furthermore, other factors affecting overall procedure time and completion, such as bowel preparation and procedure difficulty, were not documented. Although not documented, these factors are assumed to be equally distributed in the two groups owing to randomization effects and, therefore, are not assumed to affect our primary outcome. Similarly, the study would have benefitted from measuring the duration of every procedure step, making it clear where the actual time savings occurred. In addition, recording of MOAA/S every 5 minutes may be imprecise, because it was possible for patients to reach a higher or lower MOAA/S score between doses. The MOAA/S score is also more susceptible to subjectivity than other scoring systems such as evoked potentials.

Clinical advantages of remimazolam are promising with regard to other unpleasant endoscopic procedures, including gastroscopies, endoscopic ultrasound scanning, and endoscopic retrograde cholangiopancreatography, where routine use of remimazolam would be of great benefit and could be implemented after appropriate research in these scenarios. Finally, remimazolam, with a safety profile comparable to that of midazolam, could replace midazolam for improved sedation in countries where use of propofol sedation is restricted to the anesthesiologist. Remimazolam could also be an alternative for patients who are not eligible for nurse-administered propofol sedation (NAPS) due to a high American Society of Anesthesiology score, sleep apnea, or other limitations. Lastly, the price of remimazolam also must be considered, because it is currently approximately 20 times higher than that of midazolam.

## Conclusions

This study demonstrates the superiority of the combination of remimazolam with fentanyl over midazolam with fentanyl for conscious sedation during colonoscopy among FIT-positive screening participants, resulting in shorter procedure time, less postoperative need for observation, lower patient pain scores, and higher satisfaction.

## Supplementary Material

Supplementary Material Zusatzmaterial
