# Supplementary material for: Efficacy of remimazolam with fentanyl vs midazolam with fentanyl for sedation in screening colonoscopy: Randomized controlled study
Source: Endosc Int Open. 2025 Aug 15;13:a26551083. doi: 10.1055/a-2655-1083 (PMC12371649; doi:10.1055/a-2655-1083)
Supplement: Supplementary file 1 — Supplementary Material [file 10-1055-a-2655-1083_26585848.pdf]

Supplementary Table 1 Description of Modified Observer's Assessment of Alertness/Sedation scores (MOAA/S).

Score	Description
5	Responds readily to name spoken in normal tone
4	Lethargic response to name spoken in normal tone
3	Responds only after name is called loudly and/or repeatedly
2	Responds only after mild prodding or shaking
1	Responds only after painful trapezius squeeze
0	No response after painful trapezius squeeze

Supplementary Table 2 Standard discharge criteria.

**Discharge criteria**<br>(Scoring of patients before and after the procedure)		
**Parameter**	**Score**	**Criteria**
Consciousness		
	0	Awake
	1	Kan be awakened by normal tone of voice
	2	Can be awakened by gentle stimuli (e.g., blood pressure measurement)
	3	Cannot be awakened by gentle stimuli
Respiratory rate		
	0	12-20/min
	1	9-11/min or over 20/min
	2	0-8/min or more than 3 L/min oxygen needed
Oxygen saturation without supplemental oxygen		
	0	> 92%
	1	90%-92%
	2	< 90%

Discharge criteria
The patient can be discharged by nursing staff with a total score of 0-1 unless the patient was in a stable condition with a higher score before the procedure. In case of doubt, always contact the responsible physician. Patients with a score < 1 can be discharged by the responsible physician. Alternatively, patients should be observed in the recovery room every 15 minutes with documentation until a total score of 0-1 is achieved.
